# Neuropsychiatric manifestation of confusional psychosis due to Cryptococcus neoformans var. grubii in an apparently immunocompetent host: a case report

**DOI:** 10.1186/1757-1626-2-9084

**Published:** 2009-11-25

**Authors:** Peralam Yegneswaran Prakash, Rao P Sugandhi

**Affiliations:** 1 Department of Microbiology, 2nd Floor, Centre for Basic Sciences Building, Kasturba Medical College, Manipal University, Madhav Nagar, Manipal, Karnataka, 576104, India

## Abstract

Cognitive disorders like dementia, delirium, depression, anxiety, psychosis and mania are the commonest neuropsychiatric manifestations. We discuss here a case of an adult women presenting with neuropsychiatric manifestations of confusional psychosis owing to Cryptococcosis. The principal cause was consequently established by culturing *Cryptococcus neoformans *from the cerebrospinal fluid confirmed as *C. neoformans *var. *grubii *(Serotype A) by genotypic methods. Antifungal therapy with IV Amphotericin B lead to sustained improvement and recovery of the patient from behavioural disorders. The case discussed here invokes the need for the vigilance it demands in delineating organic brain syndromes for a favourable treatment outcome.

## Introduction

Among the vast diversity of uncanny neuropsychiatric manifestations a minor section is attributed to agents of infectious diseases. The involvement *of Cryptococcus neoformans *as a causal agent in mounting behavioural disorders like psychosis is of rare occurrences [1]. Human Cryptococcosis often presents with diverse spectrum of manifestations ranging from pulmonary, central nervous system, cutaneous, osseous and occasionally with potential dissemination in immunocompromised hosts [2]. The central nervous system involvement leading to meningitis invariably ends in fatality if untreated. In the setting of a developing country the victims of psychiatric ailments are brought in for an expert medical intervention only as the symptoms aggravate. Thus the patient often suffer owing to the extend course of the disease. The Indolent progression of Cryptococcosis due to *C. neoformans *var. *grubii *in an apparently immunocompetent patient leading a protracted course of clinical manifestation as confusional psychosis is discussed in this report.

## Case Presentation

A 28-year-adult woman hailing from a rural area in India was admitted to the psychiatric unit of our tertiary case hospital with complaints of abrupt and dwindling mental status in the preceding 3 months. She presented with noticeable behavioural changes characterized by brief episodes of confusion, hallucination, delusion and psychosis. Patient was unable to distinguish reality from fantasy. There was neither a family history psychiatric ailments nor a past history of diagnosed behavioural disorder in the patient. Upon examination, the muscle tone and brain CT scan and EEG were normal and she was not photophobic. A tentative mood disorder was diagnosed and patient was started on Olanzapine 5 mg/day.

But a gradual deterioration in the patient mental status occurred during the 4^th ^day of admission with periodic episodes of fever. At this time a lumbar puncture was performed with a recorded opening pressure of 120 mm. The CSF was faintly turbid with a protein content of 1.25 g/l, sugar 1.8 mmol/l and 4-5 WBC/ml. A portion of CSF sample send to perform microbiological investigations turned out to be negative for bacterial and viral agents including HIV tests. The CSF microscopy examination using India Ink preparation was negative for Cryptococcus. A CSF Culture for Cryptococcus was carried out which on prolonged incubation up to 1 week revealed a few colonies of Cryptococcus. Biochemical and physiological tests were performed as per standard mycological methods and the isolate presumptively identified as *Cryptococcus neoformans*. Subsequently the genotyping results confirmed the isolate being C. *neoformans *var. *grubii*.

A confirmed diagnosis of meningitis due to *Cryptococcus neoformans *was made and patient shifted to the General medicine unit. Treatment with IV Amphotericin B initiated and the psychotropic medicaments discontinued. The patient retained mental stability at the end of 12 weeks and was discharged from the hospital. Upon follow up after a year the patient remained well with no recurrence of psychosis.

## Discussion

The initial presentation of behavioural disorder in our patient had made the patients caretakers and local therapists believe it to be linked with an event concerned with devilish possession. Subsequently the mycological diagnosis leading to adequate antifungal treatment could bring about complete resolution of psychotic symptoms.

Summary of the patient demographic features with the typical presenting signs and symptoms, definitive diagnostic test and outcome is tabulated in [Table 1].

**Table 1:** Reported cases of neuropsychiatric manifestations due to *Cryptococcus neoformans*

Reference	Age, Gender	Location	Symptoms	Immune status	Definitive diagnostic test	Treatment/Outcome
**Present case**	28, Female	India	Insomnia, Irritability, Psychosis, impaired reality testing, confusion	Immune-competent	CSF Culture	Amphotericin B; Recovery after 12 weeks
**Ref. **[3]	25, Female	China	Elated mood, logorrhoea, flight of ideas, disorientation,	Not mentioned	CSF Culture, Cryptococcal antigen test	Amphotericin B; Recovery after 16 weeks
**Ref. **[4]	63, Male	USA	Insomnia, restless, irritable, agitation, social dis-inhibition	Not mentioned	CSF Culture, Cryptococcal antigen test	Amphotericin B; Recovered
**Ref. **[5]	32, Male	USA	Insomnia, auditory and visual hallucinations, paranoid delusions	Immune-compromised	CSF (India Ink) Blood culture	Recovered
**Ref. **[5]	35, Male	USA	Poor sleep, Intrusive, hyperactive	Immune-compromised	CSF (India Ink)	Died
**Ref. **[6]	38, Male	Papua New Guinea	Restlessness, elated mood, delusions	Immune-competent	Serum Cryptococcal antigen test	Died
**Ref. **[7]	27, Male	France	Hallucinations, Delusional ideas	Immune-compromised	CSF (India Ink) Culture	Recovered
**Ref. **[8]	22, Male	Kuwait	Confusion Psychosis	Not mentioned	CSF Culture	Amphotericin and 5-flurocytosine; Recovered
**Ref. **[9]	57, Male	Israel	Vascular dementia	Immune-competent	CSF (India Ink) Culture Serum and CSF Cryptococcal antigen test	Amphotericin B, 5-Flucytosine followed by Flucanazole; Recovered

Although there is no significant influence on the clinical outcome based on the varietal types of *Cryptococcus neoformans*, often *C. neoformans *var. *gattii *has been found to have involved in immunocompetent host [3]. On the other hand in the present case the agent involved was *C. neoformans *var *grubii *and is the first report from southern state of India.

It is interesting to note the lack of pathognomonic radiological features made the confirmed diagnosis a challenging endeavour. In addition psychosis and associated behavioural disorder is tempted to over look the true aetiology leading to misdiagnosis in the early stage. It is difficult to pinpoint how much of the resolution was due to the prior antipsychotic drug since it was discontinued as soon as the mycological diagnosis of cryptococcosis was confirmed. The existing literature has very few information on the evidence for the number of cases in which *Cryptococcus neoformans *may be involved with psychotic manifestation. In this case where the antipsychotic treatment was discontinued and the patient was put on antifungal treatment for duration of 12 weeks there was complete cure and we implicate the pathogen role in the root cause for the psychoses.

In earlier literature describing neuropsychiatric manifestation of cryptococcosis the varietal confirmation of the *Cryptococcus neoformans *isolates has not been carried out [4-10]. In this present case we have attempted to provide this date by genotyping the isolate. This could be important as the variety types has different ecological, epidemiology and virulence properties and will help us to understand the diverse presentation of cryptococcosis in a wider population especially in a scenario where the immune status of the host is difficult to determine.

Too frequently a psychiatric diagnosis is given prematurely and often an aggressive thought of infections as a differential diagnosis is given less priority. The case discussed here invokes the need for the vigilance it demands in delineating organic brain syndromes in significantly reducing the mortality with complete recovery from the neuropsychiatric manifestations.

## Consent

The patient deteriorated mental status made the informed consent procedure reasonably difficult. To this extent consent was obtained from the patient's next-of-kin and effort has been made so that patient identity remains anonymous and there is no reason to think that the patient or their family would object to publication.

## Competing interests

The authors declare that they have no competing interests.

## Authors' contributions

PYP followed up the case, did the literature review and drafted the manuscript. PSR analysed the case. Both authors read and approved the final manuscript.
